# Indoxyl Sulfate Down-Regulates SLCO4C1 Transporter through Up-Regulation of GATA3

**DOI:** 10.1371/journal.pone.0066518

**Published:** 2013-07-09

**Authors:** Yasutoshi Akiyama, Koichi Kikuchi, Daisuke Saigusa, Takehiro Suzuki, Yoichi Takeuchi, Eikan Mishima, Yasuaki Yamamoto, Ayako Ishida, Daiki Sugawara, Daisuke Jinno, Hisato Shima, Takafumi Toyohara, Chitose Suzuki, Tomokazu Souma, Takashi Moriguchi, Yoshihisa Tomioka, Sadayoshi Ito, Takaaki Abe

**Affiliations:** 1 Department of Community Health Promotion, Tohoku University Graduate School of Medicine, Sendai, Japan; 2 Division of Nephrology, Endocrinology, and Vascular Medicine, Tohoku University Graduate School of Medicine, Sendai, Japan; 3 Laboratory of Oncology, Pharmacy Practice and Sciences, Graduate School of Pharmaceutical Sciences, Tohoku University, Sendai, Japan; 4 Department of Medical Chemistry, Tohoku University Graduate School of Medicine, Sendai, Japan; 5 Division of Medical Science, Tohoku University Graduate School of Biomedical Engineering, Sendai, Japan; 6 Department of Clinical Biology and Hormonal Regulation, Tohoku University Graduate School of Medicine, Sendai, Japan; University of Sao Paulo Medical School, Brazil

## Abstract

The accumulated uremic toxins inhibit the expression of various renal transporters and this inhibition may further reduce renal function and subsequently cause the accumulation of uremic toxins. However, the precise mechanism of the nephrotoxicity of uremic toxins on renal transport has been poorly understood. Here we report that indoxyl sulfate, one of the potent uremic toxins, directly suppresses the renal-specific organic anion transporter SLCO4C1 expression through a transcription factor GATA3. The promoter region of SLCO4C1 gene has several GATA motifs, and indoxyl sulfate up-regulated GATA3 mRNA and subsequently down-regulated SLCO4C1 mRNA. Overexpression of GATA3 significantly reduced SLCO4C1 expression, and silencing of GATA3 increased SLCO4C1 expression vice versa. Administration of indoxyl sulfate in rats reduced renal expression of slco4c1 and under this condition, plasma level of guanidinosuccinate, one of the preferable substrates of slco4c1, was significantly increased without changing plasma creatinine. Furthermore, in 5/6 nephrectomized rats, treatment with oral adsorbent AST-120 significantly decreased plasma indoxyl sulfate level and conversely increased the expression of slco4c1, following the reduction of plasma level of guanidinosuccinate. These data suggest that the removal of indoxyl sulfate and blocking its signal pathway may help to restore the SLCO4C1-mediated renal excretion of uremic toxins in CKD.

## Introduction

Chronic kidney disease (CKD) is a global health problem that carries a substantial risk for cardiovascular morbidity and death [1]. With the progression of CKD, various uremic toxins accumulate, subsequently causing renal damage and hypertension [2], [3]. Recently, we have revealed that human kidney-specific organic anion transporter SLCO4C1 excretes uremic toxins, and the up-regulation of SLCO4C1 resulted in the reduction of blood pressure and renal inflammation in a CKD model [4], [5].

It has been reported that the expression levels of various transporters change in the renal failure [6], [7], and the expression level of SLCO4C1 expression is also down-regulated in the renal failure [5], [8]. Some potential factors (i.e. proinflammatory cytokines and uremic toxins) have been proposed to be involved in their expression changes [8]–[12]. However, the down-regulation mechanism of SLCO4C1 in the renal failure has not been well elucidated.

Here we investigated the toxic potentials of various uremic toxins identified previously by our capillary electrophoresis-based MS analysis (CE-MS) [13] and identified that indoxyl sulfate (IS) directly inhibits the expression and function of SLCO4C1 through up-regulation of GATA3 transcription factor.

## Materials and Methods

### Materials

GATA inhibitor K-7174 was provided by Kowa Co. Ltd. (Tokyo, Japan). Oral adsorbent AST-120 (Kremezin®) was provided by Kureha Corporation (Tokyo, Japan). All the other compounds were purchased from Sigma-Aldrich (St. Louis, MO).

### Cell culture

Human renal carcinoma cell line ACHN and renal proximal tubule cell line HK-2 were purchased from ATCC. ACHN was grown in RPMI 1640 with heat-inactivated 10% fetal bovine serum (FBS, GIBCO Invitrogen, Grand Island, NY). HK-2 was grown in Renal Epithelial Cell Growth Medium, REGM™, Lonza, Basel, Switzerland. All cells were cultured at 37°C, 95% ambient air and 5% CO_2_.

Albumin-containing medium was prepared by dissolving bovine serum albumin (Sigma-Aldrich) at final concentration of 4% in the medium.

### Measurement of free and total concentrations of IS

IS concentration in the medium was measured by LC/MS/MS as previously reported [14]. For measuring free IS concentration, proteins including albumin in the cultured medium was removed by ultrafiltration using Ultrafree-MC, Centrifugal Filter Devices (5,000 NMWL Filter Unit, Millipore Co., Billerica, MA) according to the manufacturer's instruction, and then flow-through was subjected to LC/MS/MS analysis. For measuring total IS concentration, cultured medium was directly subjected to LC/MS/MS analysis without removing proteins.

### Western blots

Cells were lysed in RIPA buffer (Santa Cruz Biotechnology, Santa Cruz, CA). Twenty micrograms of protein were subjected to SDS-PAGE and then transferred to PVDF membranes (Immun-Blot, Bio-Rad Laboratories, CA). Blots were blocked by 5% skim milk in PBS containing 0.1% Tween 20 (PBS-T) at room temperature for 1 hour, and then probed with anti-human GATA3 antibody (Santa Cruz) or anti-human SLCO4C1 antibody (Santa Cruz) at 4°C overnight. The blots were washed and then incubated with anti-rabbit HRP-conjugated secondary antibody (PIERCE, Rockford, IL). ECL plus chemiluminescent system (GE Healthcare, Piscataway, NJ) was used for detection. For Western blotting of rat slco4c1, the crude membrane fraction of the kidney was prepared as described [15].

### Immunohistochemistry

Paraffin-embedded rat kidney sections were deparaffinized in xylene. After washing in PBS, the sections were blocked for 15 min with 10% of goat serum, then incubated overnight with anti-SLCO4C1 antibody (Santa Cruz) at 1∶200 dilution at 4°C. The sections were further processed according to the manufacturer's protocol for Histofine simple stain rat MAX-PO(R) kit (Nichirei, Tokyo, Japan) and visualized by 3,3′-diaminobenzidine tetrahydrochloride and counterstained with hematoxylin as previously reported [4].

### GATA3 over-expression

Human GATA3 cDNA was obtained from Kazusa DNA Research Institute (pF1KB8362, Kisarazu, Chiba, Japan) and the open reading frame was sub-cloned into the mammalian expression vector (pFC14K, Promega, Madison, WI). ACHN cells were transfected with the GATA3-expressing plasmid with Lipofectamine 2000 (Invitrogen, Carlsbad, CA) in accordance with the manufacturer's instruction. After 48 hours of transfection, quantitative real-time PCR analysis was performed.

### Knockdown of GATA3

ACHN cells were transfected with the gene-specific (HSS142154, Invitrogen, Carlsbad, CA) or negative control (12935–112, Invitrogen) siRNA using Lipofectamine 2000 by reverse transfection in accordance with the manufacturer's instruction. The cells were then collected at different time intervals (36, 48, 60, 72 hours later) and then subjected to Western blot and quantitative real-time PCR.

### Quantitative real-time PCR

Human quantitative real-time PCR of GATA2 (Hs00231119_m1), GATA3 (Hs00231122_m1), SLCO4C1 (Hs00698884_m1) and GAPDH (Hs99999905_m1) was performed using the TaqMan Gene Expression Assay (Applied Biosystems, Foster City, CA) in accordance with manufacturer's instruction using StepOnePlus real-time PCR system (Applied Biosystems).

### Reporter gene assay

The human 5′ SLCO4C1 promoter region (−129 bp, −504 bp and −3886 bp) were amplified by PCR and inserted into the pGL3 basic luciferase expression vector (Promega, Madison, WI). Two micrograms of plasmid and 0.1 μg of Renilla Luciferase Reporter Vector pRL-TK (Promega) were co-transfected into ACHN cells. Twenty-four hours later, the medium was changed into a medium with or without 10 μM of K-7174. And 24 hours after the change of the medium, reporter assay was performed using Dual Luciferase Reporter Assay System (Promega).

### Animal studies

For IS administration, SD rats at the age of 6 weeks were obtained from Charles River (Kanagawa, Japan). At the age of 7 weeks, rats were divided into two groups: control group (n = 5) and IS group (n = 5). The IS group was given 0.1% indoxyl sulfate potassium salt (Biosynth Chemistry & Biology, Staad, Swizerland) dissolved in drinking water for 4 weeks.

For AST-120 administration, Wistar rats were obtained from Charles River. At age 9 weeks, five-sixths nephrectomy (5/6Nx) or sham operation was performed as previously reported [4]. 10 weeks after the operation, 5/6Nx rats with creatinine clearance in the range of 1.0–2.5 ml/min were divided into two groups. Then, the AST-120 group (n = 15) was fed powder chow (CE-2, CLEA Japan, Inc., Tokyo, Japan) containing 8% (w/w) of AST-120 for 4 weeks, whereas the sham-operated (n = 6) and control (n = 17) groups were fed powder chow alone. All the animal experiments were approved by the Center for Laboratory Animal Research, Tohoku University.

### Blood and urine analysis

Plasma creatinine was measured with i-STAT (Abbott Point of Care Inc., Princeton, NJ) [16], [17]. Urinary creatinine was measured enzymatically (SRL Inc., Tokyo, Japan). Plasma free IS was measured by high-performance liquid chromatography. Plasma guanidinosuccinate, ADMA and *trans*-aconitate were measured by LC/MS/MS as previously reported [14].

### Statistics

The data were expressed as means ± SEM. Student's *t*-test was performed for comparison between two groups. Analysis of variance (ANOVA) was performed for comparison among groups. P<0.05 was considered to be significant.

## Results

### Effects of uremic toxins on the SLCO4C1 expression

**Figure 1:**
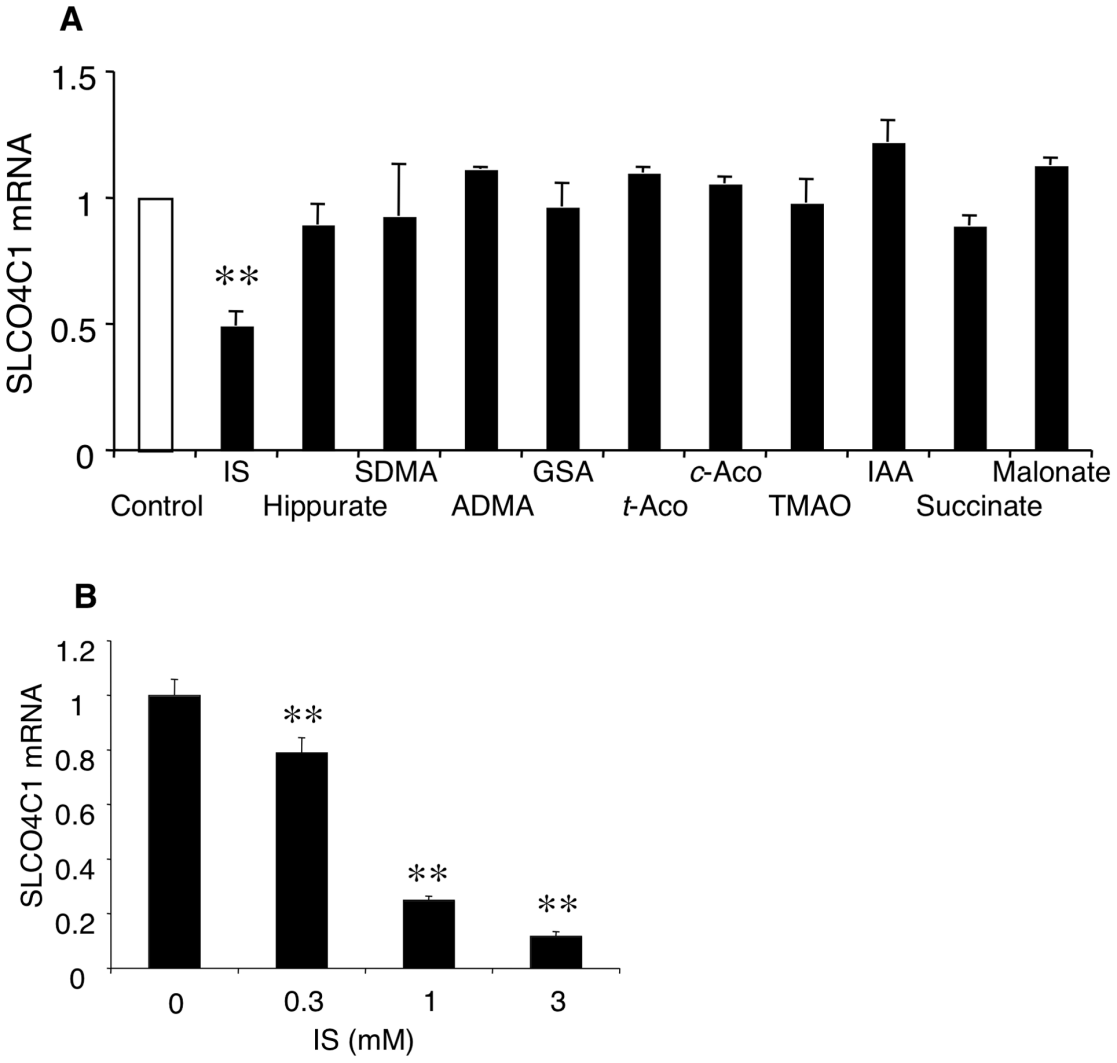
Effects of IS on SLCO4C1 expression. (A) The mRNA expression levels of human SLCO4C1 24 h after treatment with various uremic solutes in ACHN are shown. IS, indoxyl sulfate; SDMA, symmetric demethylarginine; ADMA, asymmetric dimethylarginine; GSA, guanidinosuccinate; t-Aco, *trans*-aconintate; c-Aco, *cis*-aconitate; TMAO, trimethylamine *N*-oxide; IAA, indole-3-acetate. All the concentrations were 1.0 mM. (B) Effect of IS on SLCO4C1 mRNA expression in HK-2 cells. HK-2 cells were incubated with IS for 24 h. **P<0.01 versus control (n = 3–4 per group).

To identify that uremic toxin(s) directly inhibit the SLCO4C1 expression, we first examined the effect of various uremic toxins on SLCO4C1 mRNA expression *in vitro*. We have previously identified 52 compounds that accumulated significantly with the decrease of estimated glomerular filtration rate (eGFR) in CKD patients by CE-MS [13] . Among them, we examined the effect of commercially available compounds on the SLCO4C1 mRNA expression in ACHN cells at the concentration of 1.0 mM. As a result, IS significantly decreased the SLCO4C1 mRNA expression level in ACHN cells (Figure 1A). On the other hand, the other compounds did not change the SLCO4C1 mRNA. To further examine the effect of IS on SLCO4C1 expression, various concentrations of IS were applied. The mRNA expression level of SLCO4C1 was significantly decreased by IS at the concentrations of 300 μM and 1.0 mM in a dose-dependent manner (Figure 1B). Because it was previously reported that mean serum concentration of IS in CKD patients on hemodialysis were 250mM [18], it is suggested that IS decreases the SLCO4C1 mRNA expression level at a pathological concentration.

**Figure 2:**
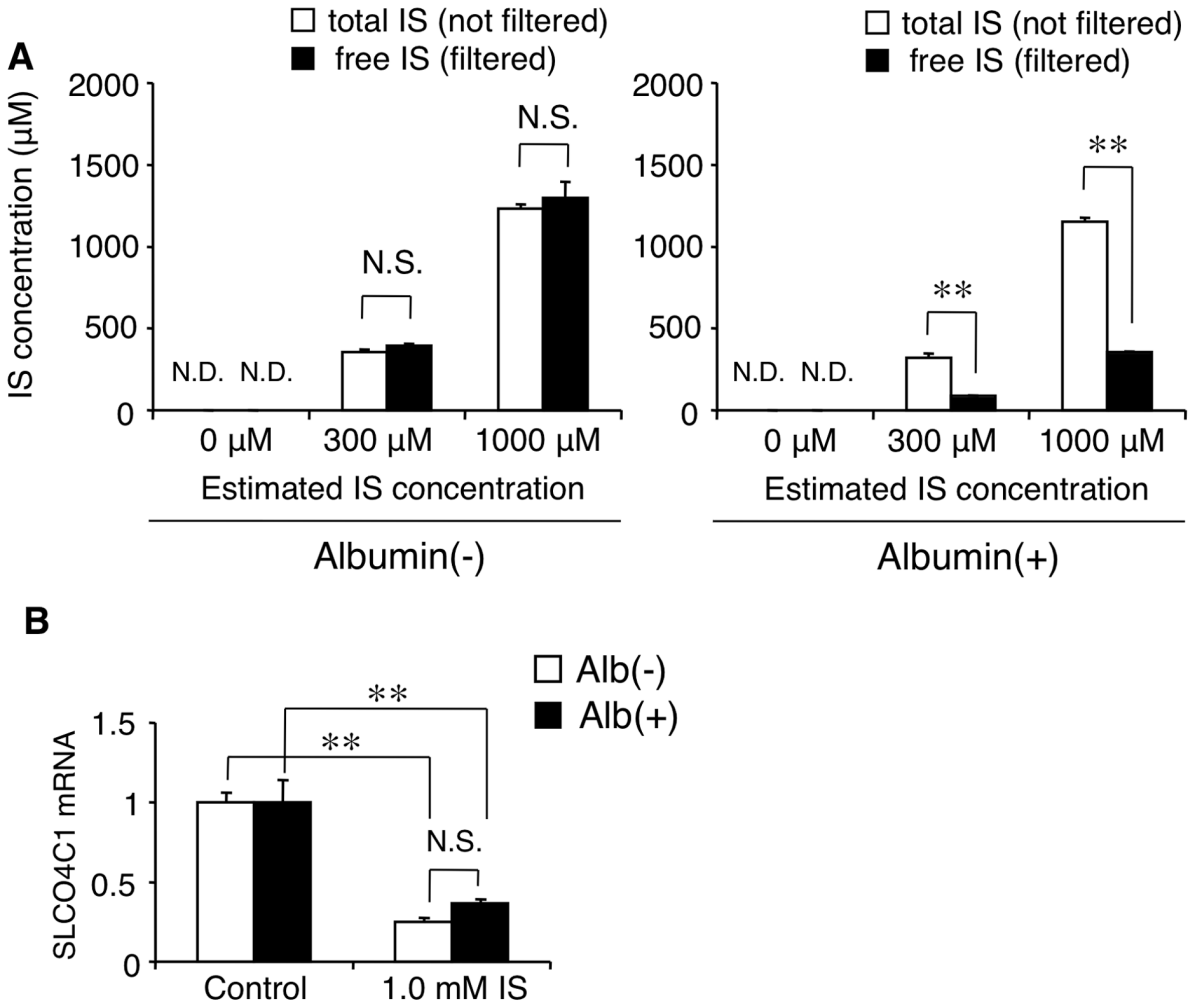
Effect of albumin on the inhibitory effect of IS on SLCO4C1 expression in HK-2 cells. (A) Total and free IS concentrations in the medium with and without albumin (n = 3 per group). (B) The inhibitory effect of IS on SLCO4C1 expression with and without albumin. **P<0.01 versus control (n = 4 per group). N.S. indicates not significant. N.D. indicates not detected.

IS is well known as a protein-bound uremic toxin [19]. To elucidate whether free fraction and/or protein-bound fraction of IS has the inhibitory effect on the SLCO4C1 expression, we next examined the effect of albumin on the inhibitory effect of IS on SLCO4C1 mRNA expression at a physiological concentration of 4% [20]. First, we measured the free and albumin-bound fraction of IS in the albumin-containing medium. In the medium without albumin, the total IS concentration and the free IS concentration were at the same level (Figure 2A). However, in the albumin-containing medium, the free IS concentration was significantly reduced compared with the total IS concentration, indicating that the free IS fraction was about 30% and 70% of IS was bound to albumin (Figure 2A). Next we evaluated the influence of albumin on the inhibitory effect of IS at the concentration of 1.0 mM. Under the condition, free IS concentration in the albumin-containing medium was estimated at about 0.3 mM (Figure 2A). After the incubation in the albumin-containing medium, the inhibitory effect of IS on SLCO4C1 mRNA expression was not statistically different from that in the medium without albumin (Figure 2B). In addition, the inhibitory effect of 1.0 mM IS with 4% albumin was much stronger than that of 0.3 mM IS without albumin (i.e. about 60% reduction and 20% reduction, respectively) (Figure 2B and Figure 1B). These data suggest that protein-bound IS as well as free IS had an inhibitory effect on SLCO4C1 expression, and that the strength of the inhibitory effect might depend on the total IS concentration, not on the free IS concentration.

### SLCO4C1 is regulated by GATA pathway

**Figure 3:**
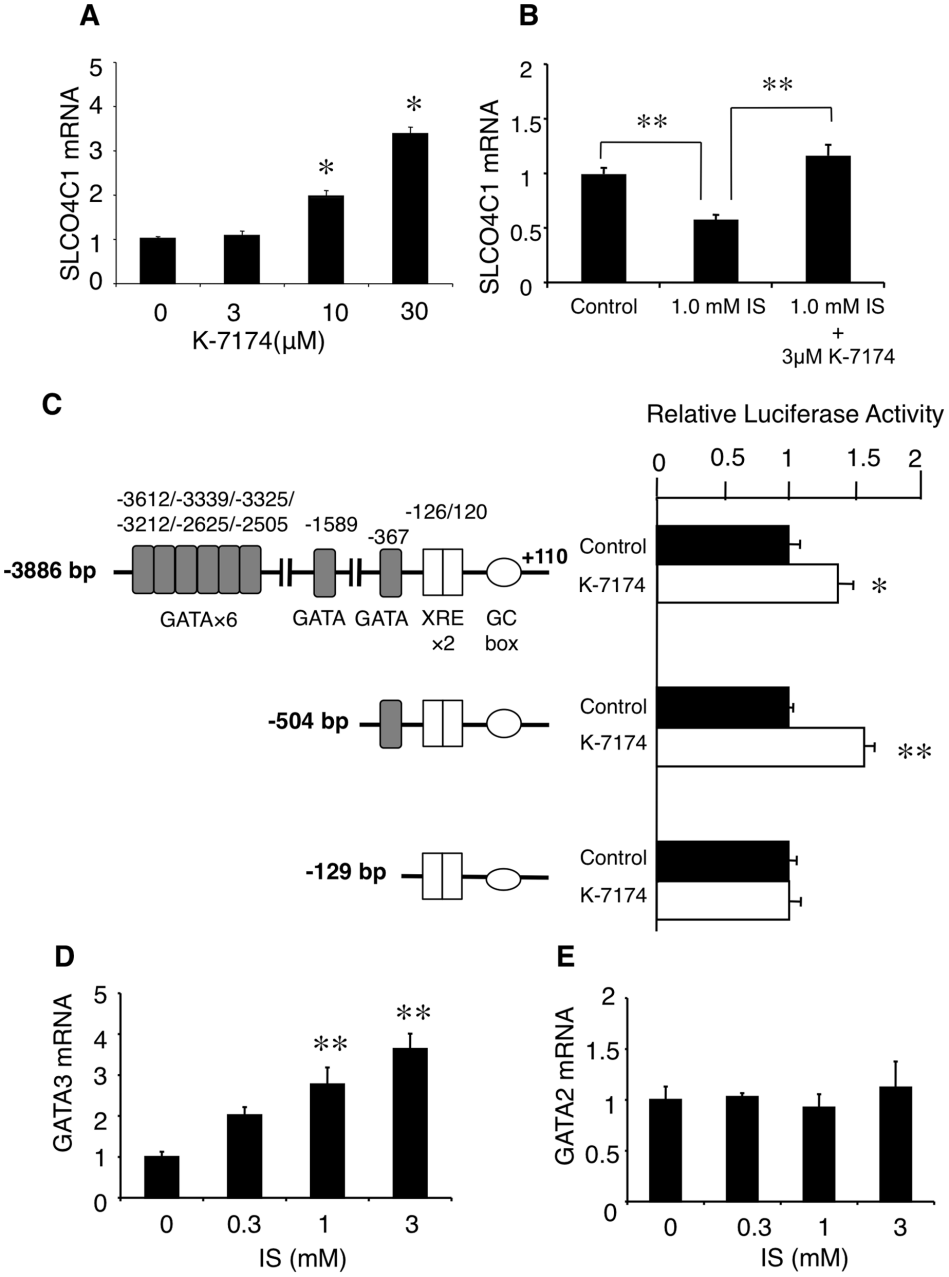
Regulation of SLCO4C1 by GATA transcriptional factor(s). A) Dose-dependent enhancement of the SLCO4C1 mRNA by K-7174 in ACHN cells. (B) Cancellation of the inhibitory effect of IS by K-7174. (C) Effect of K-7174 (10 μM) on the promoter activity of human SLCO4C1. (D and E) Effects of IS on the mRNA expressions of GATA3 (D) and GATA2 (E). *P<0.05 versus control; **P<0.01 versus control (n = 3–4 per group).

To elucidate the inhibitory mechanism of IS on SLCO4C1, we focused on the transcriptional regulator of human SLCO4C1 gene. Recently, we have reported that tandem xenobiotic-responsive element (XRE) motifs regulate SLCO4C1 transcription and statins up-regulate its transcription through the aryl hydrocarbon receptor (AhR)-XRE system [4]. In addition, we also identified several GATA motifs located upstream of the XRE motifs (Figure 3C). To elucidate the effect of GATA factors on SLCO4C1 transcription, we examined the effect of the GATA inhibitor, K-7174 [21] on SLCO4C1 expression. As a result, K-7174 significantly increased the expression of SLCO4C1 mRNA in a dose-dependent manner (Figure 3A). In addition, K-7174 canceled the inhibitory effect of IS on SLCO4C1 expression (Figure 3B). These data suggest the implication of GATA(s) on the SLCO4C1 expression.

We next examined whether GATA transcriptional factor(s) regulates SLCO4C1 gene expression. Because there are several GATA motifs, various lengths of the 5′ promoter region of SLCO4C1 were transfected into kidney cell and the effect of K-7174 on these promoter activities was examined. The luciferase activity of −3886 bp construct and the −504 bp construct were significantly increased by K-7174 (Figure 3C). On the other hand, the −129 bp construct which does not contain any GATA motifs showed no response to K-7174 (Figure 3C), suggesting that the SLCO4C1 gene expression was regulated through a single GATA motif located at −367 bp upstream from ATG codon.

Because GATA2 and GATA3 are predominantly expressed in the kidney and play an essential role in development [22]–[24], we next examined the effect of IS on GATA2/GATA3 expressions in HK-2 cells. By treatment of various concentrations of IS, the GATA3 mRNA was significantly increased in a dose dependent manner (Figure 3D). However, the GATA2 mRNA level was not changed (Figure 3E). These data suggest that IS regulates SLCO4C1 transcription through GATA system.

### GATA3 negatively regulates SLCO4C1 expression

**Figure 4:**
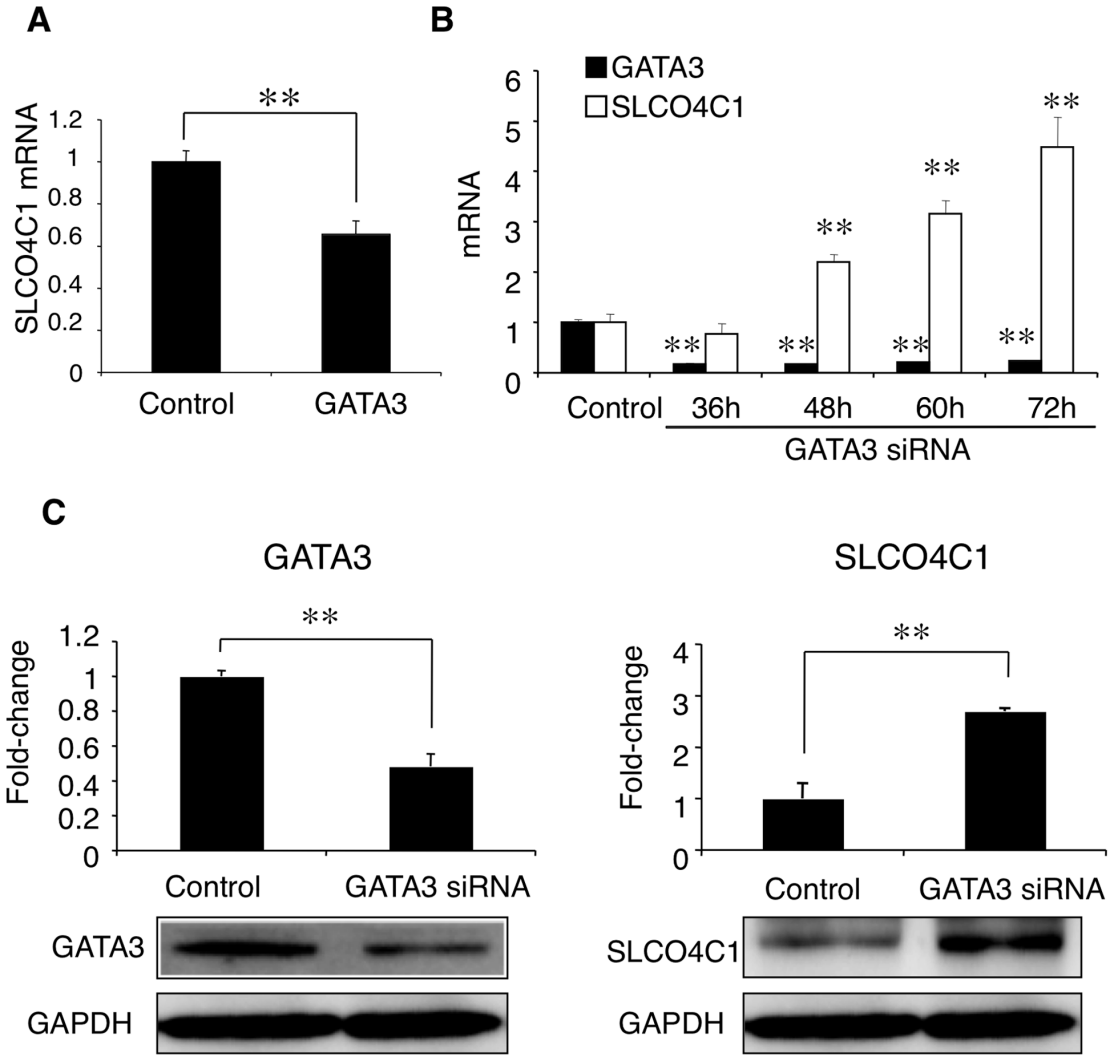
Negative regulation of GATA3 on SLCO4C1 expression. (A) Overexpression of GATA3. (B and C) Knockdown of GATA3. (B) Time-dependent alteration of the GATA3 and SLCO4C1 mRNA expression levels. (C) Western blot analysis of GATA3 (left) and SLCO4C1 (right). The bands were quantified by densitometry and normalized to the level of GAPDH. A representative band is shown. **P<0.01 versus control (n = 3 per group).

We next focused on the effect of GATA3 on SLCO4C1 expression. In ACHN cells, overexpression of human GATA3 significantly reduced the SLCO4C1 mRNA expression (Figure 4A). Knockdown of GATA3 by siRNA significantly decreased GATA3 mRNA and, SLCO4C1 mRNA was significantly increased reciprocally by down-regulation of GATA3 (Figure 4B). At the protein level, GATA3 protein was significantly decreased by GATA3 silencing (Figure 4C left) and the SLCO4C1 protein was significantly increased compared with control (Figure 4C right). These data clearly suggest that SLCO4C1 was negatively regulated transcriptionally by GATA3.

### IS decreases slco4c1 transport activity

**Figure 5:**
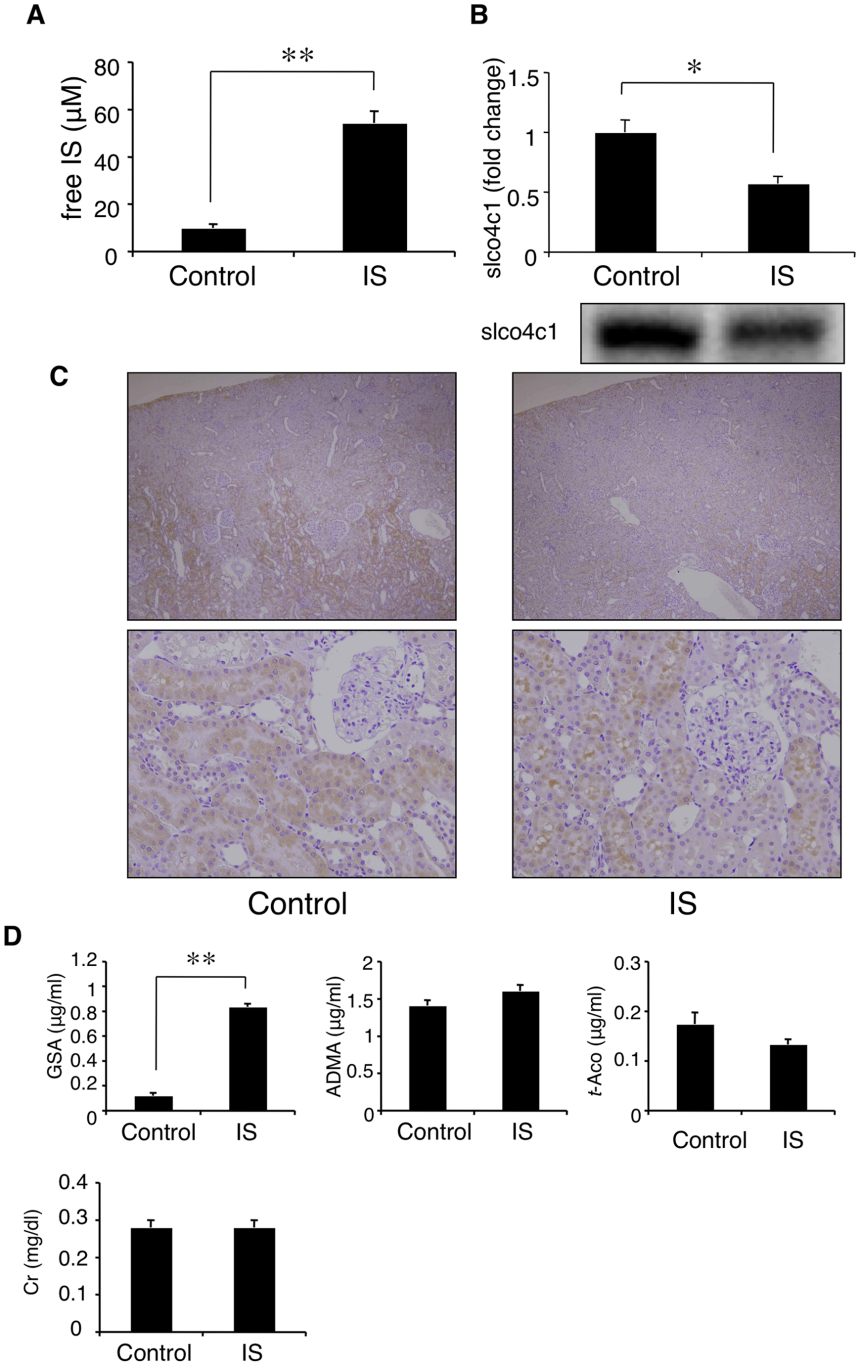
Effect of IS on slco4c1 activity *in vivo*. (A) Plasma indoxyl sulfate (IS) concentration after 4 weeks administration of IS. (B) Western blot analysis of slco4c1. (C) Immunohistochemical staining of slco4c1 in rat kidney. (D) Plasma concentrations of creatinine (Cr) and representative substrates for slco4c1 transporter, GSA, ADMA and *trans*-aconitate. *P<0.05; **P<0.01.

Based on these results, we next examined the relation of slco4c1 expression level and its function by administration of IS. After 4 weeks administration of IS, the plasma IS level was significantly increased compared with the control group (54.4 μM and 10.0 μM, respectively) (Figure 5A), and the renal protein level of slco4c1 was significantly decreased (Figure 5B). Immunohistochemical analysis also revealed that the immunostaining of slco4c1 was reduced in IS-treated kidney compared with control (Figure 5C). Under this condition, plasma GSA concentration was significantly increased, although plasma creatinine was not changed (Figure 5D). These data suggested that IS decreases renal slco4c1 expression, which may reduce the excretion of uremic toxin without change of glomerular function. Under the condition, plasma concentrations of ADMA and *trans*-aconinate were not changed (Figure 5D).

### Removal of IS increased slco4c1 expression in CKD model

**Figure 6:**
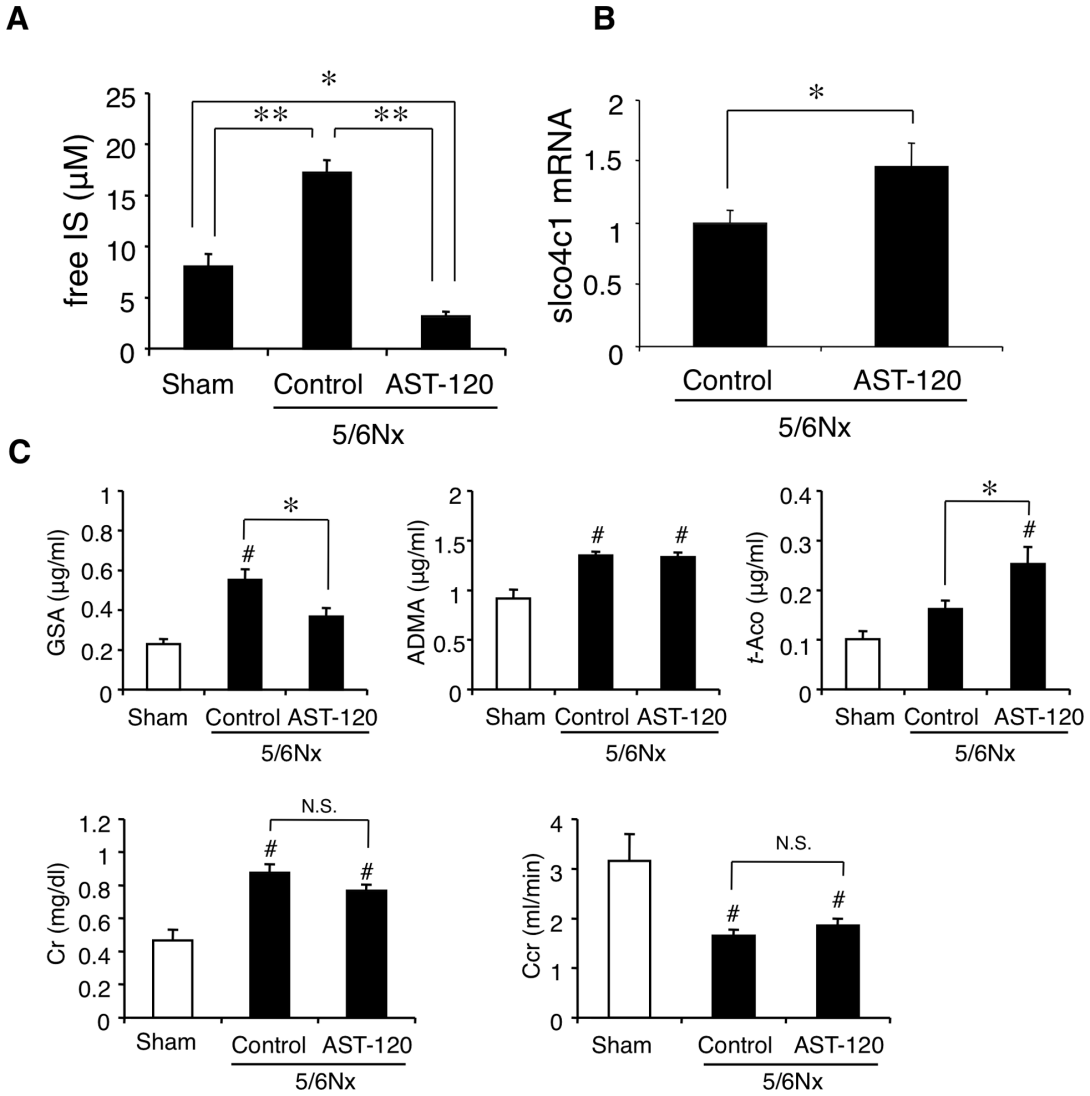
Effect of AST-120 on slco4c1 expression in the renal failure *in vivo*. (A) Plasma IS concentration after 4 weeks of treatment of AST-120. (B) mRNA level of rat slco4c1 in the kidney after AST-120 administration. (C) Plasma concentrations of creatinine, creatinine clearance (Ccr) and representative substrates for slco4c1 transporter, GSA, ADMA and *trans*-aconitate. *P<0.05; **P<0.01; ^#^P<0.01 versus control.

Clinically, oral adsorbent AST-120 has been used to remove serum IS [25]. To elucidate whether lowering the plasma IS concentration in CKD increases renal slco4c1 expression *in vivo*, oral adsorbent AST-120 was administered to subtotal nephrectomized (5/6 Nx) renal failure rats. In 5/6 Nx rats, the plasma level of IS was significantly higher compared with sham-operated group (17.2 μM and 8.0 μM, respectively). In addition, the plasma IS concentration in AST-120-administered group (3.2 μM) was significantly decreased compared with that in sham-operated and control groups (Figure 6A). The renal slco4c1 mRNA expression level in the AST-120-treated group was significantly increased compared with the control group (Figure 6B). In addition, the plasma GSA concentration was significantly decreased by AST-120 treatment (Figure 6C), although plasma creatinine and creatinine clearance were not changed between control and AST-120-treated group. These data suggest that oral adsorbent AST-120 may increase slco4c1 expression and facilitate the slco4c1-mediated excretion of uremic toxin in CKD status.

## Discussion

Here, we revealed that the uremic toxin transporter, SLCO4C1, is negatively regulated by the uremic toxin, IS through GATA3. This reduction of SLCO4C1 caused the further accumulation of uremic toxins which are excreted through SLCO4C1.

In human, SLCO4C1 is the only organic anion transporter polypeptide (OATP) expressed in the kidney and localized at the basolateral membrane of proximal tubules [5]. It is supposed that SLCO4C1 and apical MDR1 transporter cooperate to excrete the substrates from blood into urine, i.e., SLCO4C1 take them up from blood and MDR1 excrete them into urine [26]. Recently, we have also revealed that SLCO4C1 is involved in the excretion of some toxins such as GSA, ADMA and *trans*-aconitate, and up-regulation of SLCO4C1 increased the clearance of these uremic toxins in rat CKD model [4]. Because the expression of SLCO4C1 was decreased in the renal failure [5], these data suggest that the down-regulation of SLCO4C1 is one of the causes of the accumulation of uremic toxins in CKD. Because the expression of many transporters decreased in CKD rat models [6], [27], [28], our data also showed one of the mechanisms of down-regulation of transporters in CKD.

GATA transcription factors are a group of evolutionarily conserved transcriptional regulators that play critical roles in development, differentiation and cell proliferation [29], [30]. However, the physiological and pathophysiological roles of GATA factors in adult tissues, especially in the kidney, have been poorly understood. In this study, we revealed that the altered expression of GATA factor by IS is involved in the pathophysiology of CKD. It has been reported that *N* ^G^-monomethy-L-arginine(L-NMMA), an endogenous inhibitor of nitric oxide synthase(NOS), inhibited EPO gene expression by both increasing GATA2 mRNA expression and GATA binding activity [31]. However, L-NMMA was not accumulated in CKD patients and not considered as a uremic toxin [32]. Thus, our data clearly showed that uremic toxins affect the expression of GATA factor.

IS is one of the most representative uremic toxins [2], [25], [33]. So far, various toxic effects of IS have been reported, such as endothelial dysfunction [34], induction of oxidative stress [35], up-regulation of ICAM-1 and MCP-1 [36], induction of TGF-β1 [37] and NF-κB [38]. Our data suggests the further importance of IS as a therapeutic target for CKD patients. In addition, it has been reported that IS also inhibit transport activity of some transporters such as MRP4 (multidrug resistance protein 4) and BCRP (breast cancer resistance protein) [39] . Our data showed that IS can inhibit transporters transcriptionally as well as functionally. However, the mechanism that IS increased the expression of GATA factors and the effects of dysregulation of GATA factors in other organs remain unknown. Further study is needed to clearly define the pathological role of IS and GATA factors in CKD.

IS is a protein-bound uremic toxin [19]. It has been reported that the mean concentrations of total and free IS in uremic populations were 23.1 mg/L and 3.22 mg/L, respectively [40], which suggests that the protein-bound IS fraction was over 80% in CKD patients. In our experiments, free IS fraction in the albumin-containing medium was about 30% and about 70% of IS was albumin-bound (Figure 2A). Our data showed that not only free but also protein-bound IS have inhibitory effect on SLCO4C1 expression. In our *in vivo* experiments, we measured only free IS concentration. Although free IS concentrations of IS-administered rats and 5/6Nx control rats were relatively low (54.4 μM and 17.2 μM, respectively) (Figure 5A and 6A), it is estimated that total IS concentrations were much higher as described above, which is high enough to inhibit slco4c1 expression as observed *in vitro*. Our data also showed the possibility that IS causes the accumulation of uremic toxins that are substrates of SLCO4C1. However, whether IS causes the accumulation of IS itself remains unknown. IS is taken up by OAT1 (SLC22A6) and OAT3 (SLC22A8) at the basolateral membrane of proximal tubules [41], not through SLCO4C1. While it is unclear whether up-taken IS is excreted into urine through transporters at the apical membrane, it is suggested that the down-regulation of OAT1 or OAT3 may cause the further accumulation of IS. It has been reported that OAT1 and OAT3 protein levels were not changed in 5/6Nx rats [28], while another group reported that the mRNA levels of OAT1 and OAT3 were down-regulated in 5/6Nx rats [42]. In our experiment, IS administration in rats slightly, but not significantly, decreased the renal mRNA levels of OAT1 and OAT3 (data not shown). AST-120 is known to reduce the plasma IS concentration by adsorbing indole, a precursor of IS, in the intestine [25]. Thus, halting of the malignant cycle of IS by AST-120 is further supported.

In the present study, reduction of IS by the administration of AST-120 decreased the plasma concentration of guanidinosuccinate (GSA), which is one of the preferable substrates of slco4c1 (Figure 6). GSA is generally known as one of the uremic toxins belonging to guanidino compounds. So far, some toxic effects of GSA have been reported, such as eliciting pro-inflammatory effects [43], reducing endothelial repair in response to injury [44], and causing hemolysis [45]. Furthermore, GSA may be involved in the etiology of uremic encephalopathy through the activation of NMDA receptor and the inhibition of GABA_A_ receptor [46]. In our experiments, plasma concentration of GSA were increased by IS administration and decreased by AST-120 administration, correlated with the changes of renal slco4c1 expression. Our data suggest that reducing plasma GSA concentration could be a candidate for monitoring the beneficial effect of AST-120.

In our experiments, plasma concentrations of ADMA and *trans*-aconitate, which are also substrates of slco4c1, were not changed similarly to that of GSA. There are some possibilities that could explain the difference among the compounds. For example, the expression change of slco4c1 in our experiments may not have been enough to cause the change of plasma concentrations of ADMA and *trans*-aconitate. In addition, while SLCO4C1 is the only OATP expressed in the kidney in human, some oatp transporters are expressed in rodent kidney [47]. Therefore the involvement of other oatp transporters may be postulated.

**Figure 7:**
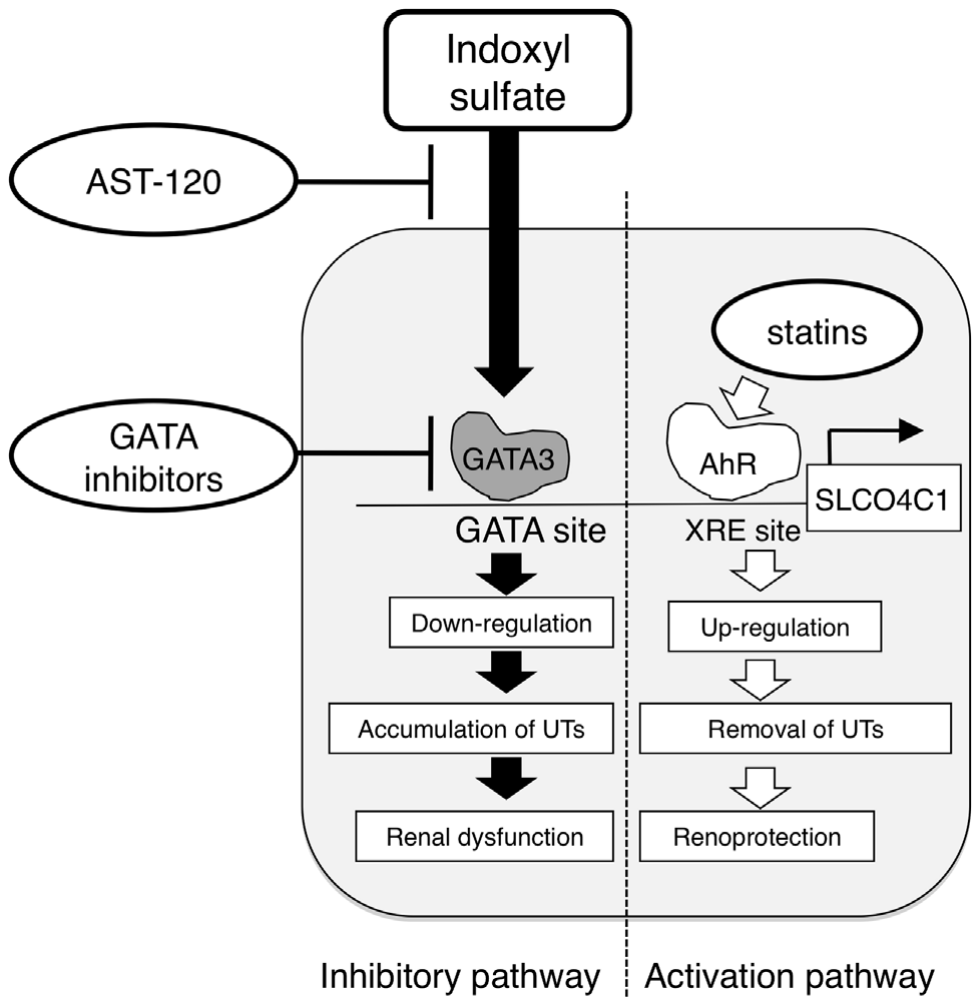
Schematic representation of putative pathological role of IS as GATA(s) inducer. IS enhances the expression of GATA factors and causes reduction of SLCO4C1 expression, followed by the further accumulation of uremic toxins, which causes the exacerbation of CKD. Oral absorbent AST-120, kidney specific GATA inhibition and up-regulation of SLCO4C1 by statins would be hopeful therapeutic strategies for CKD. UTs, uremic toxins.

Recently, we have reported that statins increase the SLCO4C1 expression and reinforce its function through AhR/XRE system [4]. In addition, the present study showed the existence of the mechanism that negatively regulates the SLCO4C1 expression. Thus, it is suggested that the expression of SLCO4C1 is dynamically regulated by the balance of activation and inhibitory pathways. Therefore, the combination therapy of, (1) activating the SLCO4C1 expression by statins, (2) reducing the plasma IS concentration by the administration of AST-120 and (3) administering GATA inhibitor to suppress GATA factors induced by IS, could be a more effective remedy for the excretion of uremic toxins and preservation of renal function in CKD patients (Figure 7).

In conclusion, it is suggested that IS plays a key role of the formation of the “vicious cycle” between the accumulation of uremic toxins and renal damage in CKD. Therefore, the removal of IS and blocking its signaling pathway should be an effective strategy to restore the SLCO4C1-mediated renal excretion of uremic toxins.
